# Doctor, when can I drive? Charakterisierung des Fahrverhaltens orthopädischer und unfallchirurgischer Patienten anhand einer prospektiven Fragebogenstudie

**DOI:** 10.1007/s00113-024-01502-5

**Published:** 2024-11-25

**Authors:** Felix Lakomek, Falk Hilsmann, Erik Schiffner, Sebastian Gehrmann, Dominique Schöps, Max Prost, Joachim Windolf, David Latz

**Affiliations:** 1 Klinik für Orthopädie und Unfallchirurgie, Universitätsklinikum Düsseldorf, Moorenstraße 5, 40225 Düsseldorf, Deutschland; 2 Klinik für Orthopädie und Unfallchirurgie, Karl-Leisner-Klinikum Kleve, Albersallee 5, 47533 Kleve, Deutschland

**Keywords:** Verkehrsmedizin, Unfallchirurgie, Kraftfahrereignung, Fahrtauglichkeit, Gelenkerkrankungen, Traffic medicine, Trauma surgery, Impaired driving function, Driving capability, Joint disorders

## Abstract

**Fragestellung:**

Die individuelle Mobilität im Straßenverkehr hat in Deutschland, sowohl individuell als auch sozioökonomisch betrachtet, einen hohen Stellenwert. Insbesondere Erkrankungen und Verletzungen im Bereich des muskuloskeletalen Systems können jedoch temporär zu Beeinträchtigungen führen. Ziel dieser prospektiven Patientenbefragung war es zu erfassen, wie die Patienten ihre Fahrtauglichkeit während einer Verletzung und damit verbundenen Ruhigstellung einschätzten und auf welcher Grundlage die Entscheidung über die Fahrtauglichkeit seitens der Patienten getroffen wurde.

**Material und Methoden:**

Anhand eines systematischen Fragebogens wurden insgesamt 100 Patienten mit einer orthopädischen/unfallchirurgischen Diagnose und einer damit verbundenen Gelenkruhigstellung analysiert. Neben persönlichen Daten und der Verletzungen/Erkrankungen wurde eine Analyse zur Risikobereitschaft durchgeführt, und die Patienten wurden zu ihrem Wissen bezüglich der Fahrtauglichkeit befragt. Abschließend wurde erfasst, welche Patienten aus welchen Gründen trotz Ruhigstellung ein Kraftfahrzeug (Kfz) geführt haben.

**Ergebnisse:**

Insgesamt gaben 40,2 % an, Kenntnis über das geltende Recht hinsichtlich der Fahrtauglichkeit zu haben. Weiterhin schätzten 55,6 % den behandelnden Arzt als Verantwortlichen bezüglich der Entscheidung über die Fahrtauglichkeit ein. Die Patienten, die ein Kfz führten, gaben eine höhere sowohl private als auch berufliche Abhängigkeit vom Kfz an (privat: 60,6 % vs. 45,7 %; beruflich: 48,5 % vs. 36,1 %). In der Patientengruppe, die ein Kfz während der Ruhigstellung führte, war insgesamt seltener eine Fraktur der Grund der Ruhigstellung (33,3 % vs. 51,0 %).

**Diskussion:**

Insgesamt schätzte das Patientenkollektiv seine Kenntnis über die Gesetzeslage als gering ein und sah den behandelnden Arzt in der Mehrzahl in der Entscheidungsverantwortung hinsichtlich der Fahrtauglichkeit. Die Patienten, die ein Kraftfahrzeug während der Ruhigstellung führten, gaben eine höhere private als auch berufliche Abhängigkeit vom Führen eines Kfz an. Gleichzeitig hatte die Verletzungsschwere einen Einfluss auf die Entscheidung, sodass Patienten mit Frakturen eine Fahrt mit einem Kfz eher vermieden. Weitere Studien insbesondere auf biomechanischer Ebene sind erforderlich, um für den Arzt eine bessere Grundlage für die Entscheidungsfindung hinsichtlich der Fahrtauglichkeit bei orthopädischen/unfallchirurgischen Erkrankungen zu gewährleisten.

**Graphic abstract:**

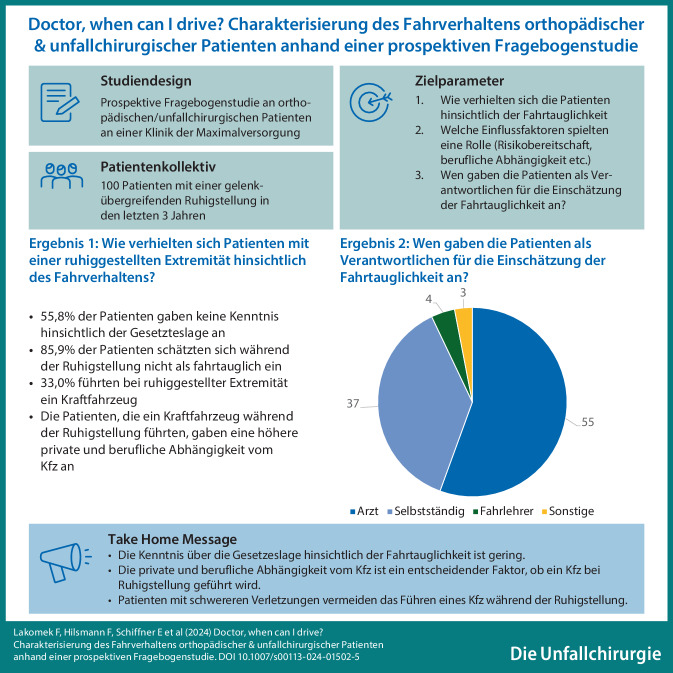

## Abstract (en)

**Background:**

Individual mobility in road traffic is of high importance in Germany, both individually and socioeconomically; however, diseases and injuries of the musculoskeletal system in particular can lead to temporary impairments. The aim of this prospective patient survey was to record how patients assessed their driving capability during an injury and the associated immobilization and on what basis the decision on driving capability was made on the part of the patients.

**Material and methods:**

A systematic questionnaire was used to analyze a total of 100 patients with a diagnosis in orthopedics/trauma surgery and associated joint immobilization. In addition to personal data and the injuries/illnesses, an analysis on risk tolerance was performed and patients were asked about their knowledge regarding driving capability. Finally, it was recorded which patients drove a motor vehicle and for what reasons despite immobilization.

**Results:**

Overall, 40.2% reported knowledge of the applicable laws regarding driving capability and 55.6% considered the treating physician to be responsible regarding the decision on driving capability. The patients who drove a motor vehicle reported higher personal and professional dependence on the motor vehicle (personal: 60.6% vs. 45.7%; professional: 48.5% vs. 36.1%). In the group of patients who drove a motor vehicle during immobilization, overall a fracture was less likely to be the reason for immobilization (33.3% vs. 51.0%).

**Conclusion:**

Overall, the patient population rated their knowledge of the law as low and viewed the treating physician as having the majority of the decision-making responsibility regarding driving capability. The patients who drove a motor vehicle during immobilization reported a higher personal as well as professional dependence on driving a motor vehicle. At the same time the injury severity had an influence on the decision, so that patients with fractures were more likely to avoid driving a motor vehicle. Further studies, particularly at the biomechanical level, are needed to ensure a better basis for the physician in making decisions with respect to the driving capability of orthopedic and trauma surgery patients.

## Einleitung

Die individuelle Mobilität im Straßenverkehr hat trotz der Mobilitätswende in Deutschland, sowohl individuell als auch sozioökonomisch betrachtet, hohen Stellenwert, kann jedoch durch Erkrankungen und Verletzungen des muskuloskeletalen Systems temporär beeinträchtigt sein. In Deutschland sind Personen als fahrgeeignet einzustufen, bei denen nach § 2 Abs. 4 StVG „die notwendigen körperlichen und geistigen Anforderungen“ erfüllt sind [8]. Die Fahreignung muss von der Fahrtauglichkeit unterschieden werden [13]. Die Fahrtauglichkeit beschreibt eine situationsbezogene und momentane Fähigkeit, ein Fahrzeug ordnungsgemäß und sicher zu führen. Verletzungen des Bewegungsapparates führen häufig zu der Frage, ob die temporäre Einschränkung der körperlichen Fähigkeiten eine Fahruntauglichkeit darstellt [28]. Neben den traumatologisch bedingten Einschränkungen wird aktuell auch die Auswirkung des alternden Patienten und der damit verbundenen Einschränkungen des Bewegungsapparates auf das Führen eines Kraftfahrzeuges (Kfz) diskutiert [27]. Gleichzeitig kann die Fahrtauglichkeit auch temporär durch eine therapiebegleitende Medikation (z. B. Analgesie) eingeschränkt sein [16].

In bestimmten Fachbereichen der Medizin bestehen klare Empfehlungen, wann nach einem medizinischen Ereignis, oder bezogen auf eine Erkrankung, ein Kraftfahrzeug wieder geführt werden kann, wie z. B. hinsichtlich epileptischen Anfällen [11, 17].

Die Entscheidung, ob ein Kraftfahrzeug geführt werden kann, im Hinblick auf ein orthopädisches oder unfallchirurgisches Krankheitsbild, stellt jedoch aktuell eine höchst individuelle und eigenverantwortliche Ermessensentscheidung des Verkehrsteilnehmers dar. Rechtlich bindende Empfehlungen liegen aktuell nicht vor. Im Zweifel kann der Verkehrsteilnehmer seinen Arzt um Rat bitten. In einer vorausgegangenen Übersichtsarbeit konnte allerdings gezeigt werden, dass nur wenige Studien zum Thema Fahrtauglichkeit im Hinblick auf orthopädische und unfallchirurgische Krankheitsbilder vorliegen [13]. Eine evidenzbasierte Entscheidungshilfe existiert für Ärzte somit nicht. Sollten Bedenken an der Fahrtauglichkeit eines Patienten vorliegen, sind Ärzte dennoch in der Pflicht dies mitzuteilen [19]. Bei der Betrachtung der Unfallstatistik könnte dies im Zweifelsfall dazu führen, dass vom behandelnden Arzt eher frühzeitig eine Fahruntauglichkeit ausgesprochen wird, um beispielsweise rechtliche Konsequenzen zu vermeiden [20, 21]. Qurashi et al. konnten beispielsweise zeigen, dass sich die Bremsreaktionskraft bereits nach 2 Tagen nach einem Hüftgelenkersatz auf den präoperativen Wert normalisierte, jedoch eine gängige Empfehlung von sechs Wochen Fahrtkarenz ausgesprochen wird [22]. Zusammenfassend wird Patienten ein hohes Maß an Eigenverantwortung zugesprochen, ohne dass sie sich an definierten Grenzwerten orientieren können. In der vorliegenden Studie sollen daher das Fahrverhalten orthopädischer und unfallchirurgischer Patienten sowie deren Risikoprofile charakterisiert werden. Besonderer Fokus liegt auf der Fragestellung, anhand welcher Parameter sich Patienten für oder gegen eine Fahrtauglichkeit unter welchen Umständen entscheiden. Mögliche Risikokonstellationen aus Verletzungsentität, sozioökonomischer Abhängigkeit vom Kfz und daraus folgende Fehlentscheidungen und Risikopotenziale durch orthopädische und unfallchirurgische Patienten im Straßenverkehr sollen hierdurch identifiziert werden.

## Material und Methodik

### Aufbau und Durchführung

In dieser prospektiven Studie wurden die Daten von orthopädischen und unfallchirurgischen Patienten, die in der Ambulanz und der Notaufnahme eines überregionalen Traumazentrums [2] in einer Großstadt behandelt wurden, erfasst. Basierend auf der ärztlichen Anamnese wurden die Patienten in die Studie eingeschlossen. Folgende Einschlusskriterien wurden definiert: 1. Vorhandensein einer zurückliegenden orthopädischen oder unfallchirurgischen Behandlung mit Ruhigstellung mindestens eines Gelenkes, 2. Vorhandensein eines Führerscheins (mindestens Führerscheinklasse B). Folgende Ausschlusskriterien wurden definiert: 1. Minderjährigkeit, 2. akute psychiatrische Erkrankungen, 3. Drogenabusus. Allen in die Studie eingeschlossenen Patienten wurde ein standardisierter Fragebogen ausgehändigt. Alle beschriebenen Untersuchungen am Menschen wurden mit Zustimmung der zuständigen Ethik-Kommission (Nr.: 2019-468), im Einklang mit nationalem Recht sowie gemäß der Deklaration von Helsinki von 1975 (in der aktuellen, überarbeiteten Fassung) durchgeführt. Von allen beteiligten Patienten liegt eine Einverständniserklärung vor.

### Datenauswertung

Es erfolgte eine anonymisierte Auswertung der standardisierten Fragebogen. In der Kategorie „*I. Persönliche Angaben“* wurden das Alter, Geschlecht, Beruf, Händigkeit und das Standbein erfragt. In „*II. Risikoprofil“* wurden die Deutsche Version des Arnett Inventory of Sensation Seeking (AISS-d) [22] sowie die Kurzskala zur Erfassung der Risikobereitschaft (R-1) [1] ausgewertet. Der AISS‑d [22] beinhaltet 20 standardisierte Fragen zur Risikobereitschaft in verschiedenen Lebensbereichen (z. B. „Ich würde gerne an fremde und entfernte Orte reisen.“), die von den eingeschlossenen Patienten mit jeweils einem bis 4 Punkten (1: trifft gar nicht auf mich zu; 4: trifft stark auf mich zu.) bewertet wurden. Anschließend wurden die Einzelwerte addiert. Die Kurzskala zur Erfassung der Risikobereitschaft (R-1) [1] dient der Selbsteinschätzung der Risikobereitschaft und kann mit 1: gar nicht risikobereit bis 7: sehr risikobereit beantwortet werden. In „*III. Kfz“* wurden die Führerscheinklasse, Fahrleistung (km/Woche), Anwendungsbereich des Kfz, Abhängigkeit vom Kfz und Kfz-Getriebeart ausgewertet. „*IV. Orthopädische/unfallchirurgische Anamnese“* beinhaltet die zugrunde liegende Diagnose der Ruhigstellung, die Ruhigstellungart, Dauer der Ruhigstellung, anderweitige Einschränkungen des Bewegungsapparates. In „*V. Fahrverhalten“* wurde ausgewertet, zum einen, ob die Patienten trotz Ruhigstellung ein Kfz geführt haben, und wer für diese Entscheidung in den Augen der Patienten verantwortlich war. Zudem zielten die Fragen auf das generelle Fahrverhalten, die Art der Nutzung des Kfz, und wie sich das Fahrverhalten im Zeitraum der Ruhigstellung verändert hat. Weitere Subgruppenanalysen wurden durchgeführt, mit der Fragestellung, inwieweit sich das Patientenkollektiv mit Fahrt während der Ruhigstellung vom Patientenkollektiv, das keine Fahrt während der Ruhigstellung durchgeführt hat, unterschied.

### Statistische Analyse

Es wurden eine deskriptive Statistik mit Darstellung der absoluten Häufigkeiten und relativen Häufigkeiten ausgewertet sowie die Standardabweichungen angegeben.

Die statistische Analyse erfolgte mithilfe von Excel® (Microsoft Corporation, Redmond, WA, USA).

## Ergebnisse

Insgesamt wurden Daten von 106 Patienten im Rahmen der Fragebogen erfasst. Bei 6 Probanden zeigte sich eine unvollständige Bearbeitung des Fragebogens, sodass diese ausgeschlossen wurden. Somit konnten in die Auswertung nach Abschluss des Studienzeitraumes 100 vollständig ausgefüllte Fragebogen einbezogen werden.

### Persönliche Angaben und Risikoprofil

Es wurden 46 Frauen und 54 Männer befragt. Das durchschnittliche Alter lag bei 39,06 Jahren (SD ± 15,18). 95 % gaben an, Rechtshänder zu sein, und 5 % Linkshänder.

**Abb. 1:**
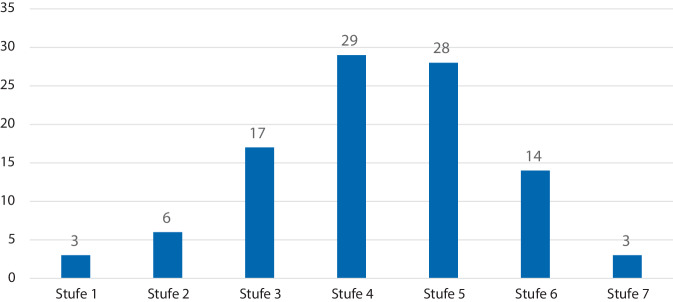
Verteilung der Risikobereitschaft (Selbsteinschätzung) der Patienten (Stufe 1 = sehr geringe Risikobereitschaft; Stufe 7 = sehr hohe Risikobereitschaft)

Die durchschnittliche Risikobereitschaft, ermittelt anhand des AISS-d (Arnett Inventory of Sensation Seeking) (8) betrug 47,8 Punkte (SD 6,24). Die Verteilung der Selbsteinschätzung in Risikogruppen ist Abb. 1 zu entnehmen. Die durchschnittliche Fahrleistung betrug beim Patientenkollektiv 255,4 km/Woche, wobei 68,8 % auf eine private Nutzung und 31,2 % auf eine berufliche Nutzung zurückfielen (Tab. 1). Die private Abhängigkeit vom Führen eines Kfz wurde mit 45,7 % und die berufliche Abhängigkeit mit 36,2 % angegeben (Tab. 1).

**Tab. 1:** Fahrverhalten und Kenntnisstand bezüglich Fahrtauglichkeit des Patientenkollektivs

*n* *=* *100*	*Mittelwert*	*Min*	*Max*	*SD*
Fahrleistung (km/w)	255,42	2	1500	272,13
Anteil privat (%)	68,8	0	100	34,44
Anteil beruflich (%)	31,2	0	100	34,44
*n* *=* *97*	*Ja (abs.)*	*Ja (%)*	*Nein (abs.)*	*Nein (%)*
Abhängigkeit privat	43	45,74	51	54,26
Abhängigkeit beruflich	34	36,17	60	63,83
*–*	*Ja (abs.)*	*Ja (%)*	*Nein (abs.)*	*Nein (%)*
Subjektive Fahrtüchtigkeit (*n* = 99)	14	14,14	85	85,86
Kenntnis der Gesetzeslage (*n* = 97)	39	40,21	58	59,79
Fahrt während der Ruhigstellung (*n* = 100)	33	33	67	67
Erkundigung über Fahrtauglichkeit (*n* = 97)	25	25,77	72	74,22
Auseinandersetzung vor Fragebogenteilnahme (*n* = 97)	41	42,27	56	57,73
Auseinandersetzung vor Verletzungseintritt (*n* = 97)	18	18,56	79	81,44

### Kfz-Art

Bei der Kfz-Art gaben 63 Patienten (63,0 %) an, einen Schaltwagen zu fahren, 23 Patienten (23,0 %) verfügten über einen PKW mit Automatikgetriebe, und 14 Patienten (14,0 %) fuhren regelmäßig beide Getriebe-Arten.

### Subjektive Fahrtauglichkeit und Fahrverhalten

**Abb. 2:**
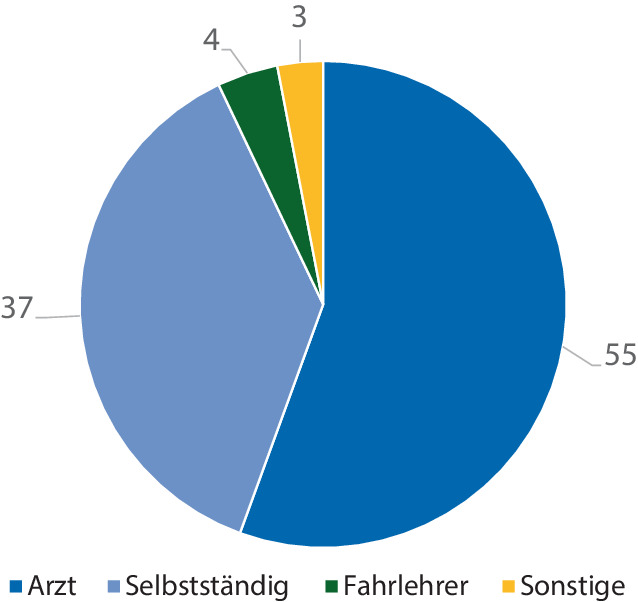
Verantwortlicher bezüglich der Einschätzung der Fahrtauglichkeit während der Ruhigstellung (abs.)

Nur ein geringer Anteil der Patienten (14,0 %) schätzte sich subjektiv als fahrtauglich während der Ruhigstellung ein, jedoch führten 33,0 % während der Zeit der Ruhigstellung ein Kfz (Tab. 1). Immerhin 40,2 % der Patienten gaben an, Kenntnis über die Gesetzeslage bezüglich der Fahrtauglichkeit zu haben, während sich nur 25,8 % der Patienten während der Ruhigstellung über die Fahrtauglichkeit erkundigten (Tab. 1). Über die Hälfte der Patienten ordnete die Einschätzung der Fahrtauglichkeit dem Arzt zu (55,0 %), ein geringerer Teil sich selbst (37,0 %) und tatsächlich einzelne Patienten ihrem Fahrlehrer (4,0 %), obwohl sie ihren Führerschein schon seit mehr als 3 Jahren besaßen (Abb. 2).

### Orthopädische/unfallchirurgische Anamnese

Bei 49 Patienten bestand eine Ruhigstellung der oberen Extremität und bei 51 Patienten eine Ruhigstellung der unteren Extremität (Tab. 2). Bei der Auswertung der ruhiggestellten Seite (rechts vs. links; Tab. 2) konnte nur eine Auswertung bei 95 Patienten erfolgen, da 5 Patienten keine Seitenangabe machten. Bei 70,0 % der Patienten war eine vollständige Ruhigstellung durch die Orthese/den Gips gegeben. Bei 30,0 % der Patienten war nur eine teilweise Gelenkruhigstellung (z. B. durch Sprunggelenkgehorthese oder Kniehartrahmenorthese) gegeben. 66 Patienten (66,0 %) gaben an, dass eine vollständige Entlastung der ruhiggestellten Extremität erforderlich war. Der häufigste Grund für die Ruhigstellung war eine Fraktur (51,0 %). 12,0 % erhielten eine Ruhigstellung aufgrund einer Distorsion, weitere 12 % aufgrund einer Bandverletzung und 25,0 % aufgrund sonstiger Verletzungen/Pathologien (u. a. Weichteilverletzung, Nervenkompressionssyndrome etc.). Die durchschnittliche Dauer der Ruhigstellung betrug 5,9 Wochen (SD 5,22). Während der Ruhigstellung haben 33 Patienten (33,0 %) des Studienkollektivs einen Pkw geführt. 19 Patienten (61,3 %) empfanden subjektiv eine mechanische Behinderung während 12 Patienten (38,7 %) keine Einschränkung durch die Ruhigstellung empfanden (Tab. 3). Als Nutzungsgrund des Kfz während der Ruhigstellung gaben 28,1 % der Probanden den Notfall an, 37,5 % eine Alltagsnutzung, 15,6 % eine berufliche Nutzung und 18,8 % eine sowohl berufliche als auch alltägliche Nutzung. Die prozentuale Fahrleistung während der Ruhigstellung lag bei 27,9 %, bezogen auf die vor der Ruhigstellung angegebenen jeweiligen wöchentlichen Kilometerleistung.

**Tab. 2:** Medizinische Angaben zur Ruhigstellung

	Abs	%
*Ruhiggestellte Extremität (n* *=* *100)*		
Obere Extremität	49	49,00
Untere Extremität	51	51,00
*Ruhiggestellte Seite (n* *=* *95)*		
Rechts	51	53,68
Links	44	46,32
*Ruhiggestelltes Gelenk (n* *=* *100)*		
Hand	4	4,00
Handgelenk	23	23,00
Ellenbogen	11	11,00
Schulter	11	11,00
Sprunggelenk	28	28,00
Kniegelenk	20	20,00
Hüfte	0	0,00
Fuß	3	3,00
*Gipsart (n* *=* *100)*		
Oberarm	10	10,00
Unterarm	23	23,00
Oberschenkel	0	0,00
Unterschenkel	6	6,00
Kein Gips	61	61,00
*Orthesenart (n* *=* *100)*		
Handgelenk	3	3,00
Ellenbogen	2	2,00
Kniegelenk	18	18,00
Sprunggelenk	20	20,00
Schulter	11	11,00
Walker-Schuh	6	6,00
Fixateur externe	1	1,00
Keine Orthese	39	39,00

**Tab. 3:** Analyse der Beweggründe und der subjektiven Erfahrung beim Führen eines Pkw mit Ruhigstellung

	Ja (abs.)	(%)	Nein (abs.)	(%)
Mechanische Behinderung subjektiv (*n* = 31)	19	61,29	12	38,71
Fahrtauglichkeit subjektiv trotz Behinderung (*n* = 20)	15	75,00	5	25,00
Üben mit Ruhigstellung vor Fahrt (*n* = 33)	3	9,09	30	90,09
Sicherheitsgefühl während der Fahrt (32)	28	87,50	4	12,50
Kfz-Art, Auswirkung auf Fahrtentscheidung (*n* = 30)	9	30,00	21	70,00

### Subgruppenanalyse

**Abb. 3:**
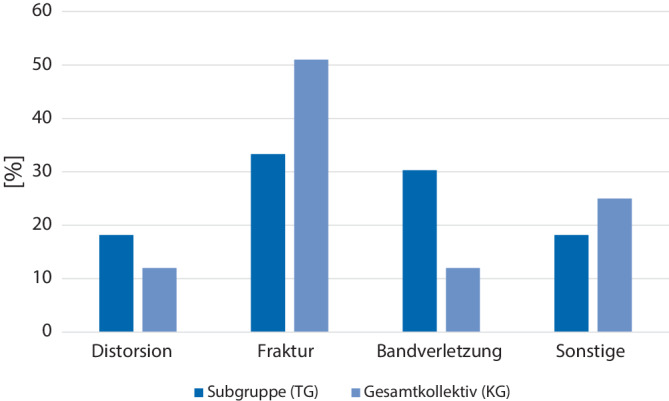
Verletzungsentitäten im Subgruppenvergleich (prozentual)

In einer Subgruppenanalyse wurden die Patienten, die während der Ruhigstellung ein Kfz (TG) geführt haben mit dem Gesamtkollektiv (KG) verglichen: Die Subgruppe wies im Vergleich ein geringeres Alter (TG = 31,6 Jahre (SD 8,91) vs. KG = 39,1 Jahre (SD 15,18)) auf. Insgesamt haben mehr Frauen ein Kfz während der Ruhigstellung im Vergleich zum Gesamtkollektiv geführt (TG = 60,6 % vs. KG = 46,0 %). Beim Vergleich der Risikobereitschaft (AISS-d) konnte kein wesentlicher Unterschied festgestellt werden (TG = 48,1 vs. KG = 47,8). Die Patienten mit Fahrt während der Ruhigstellung gaben eine höhere sowohl private als auch berufliche Abhängigkeit bezüglich des Führens eines Kfz an (Privat: TG = 60,6 % vs. KG = 45,7 %; Beruflich: TG = 48,5 % vs. KG = 36,2 %). Bei Betrachtung des Verletzungsmuster hatten die Patienten mit Fahrt während der Ruhigstellung weniger Verletzungen der oberen Extremität (TG = 33,3 % vs. KG = 49,0 %) und mehr Verletzungen der unteren Extremität (TG = 66,7 % vs. KG = 51,0 %). Häufiger war eine Ruhigstellung der linken Seite bei der Subgruppe (TG = 54,8 % vs. KG 46,3 %). Bezüglich der Verletzungsentitäten zeigten sich in der Subgruppe prozentual weniger Frakturen und dafür mehr Distorsionen und Bandverletzungen (Abb. 3). Bei der Frage nach der subjektiven Fahrtüchtigkeit schätzten sich mehr Patienten in der Subgruppe Fahrt während der Ruhigstellung als fahrtauglich ein (TG = 30,3 % vs. KG = 14,1 %).

Zudem wurde die Subgruppe mit der häufigsten Ruhigstellungsart (Sprunggelenkorthese (z. B. Aircast), *n* = 20) hinsichtlich einer Fahrt während der Ruhigstellung untersucht. 10 Patienten (50,0 %) führten ein Kfz mit anliegender Sprunggelenkorthese, während die anderen 10 Patienten eine Fahrt vermieden.

Das Handgelenk war das häufigste ruhiggestellte Gelenk (*n* = 23), hierbei führte ein nichtunerheblicher Anteil (17,4 %) der Patienten ein Kfz.

## Diskussion

Mit der vorliegenden Untersuchung zur Selbsteinschätzung der Fahrtauglichkeit und tatsächlichem Verhalten während einer Ruhigstellung nach muskuloskeletalen Verletzungen wurde nachgewiesen, wie häufig die Fahrtauglichkeit von betroffenen Patienten angenommen wurde, und auf welcher Grundlage die Patienten über ihre Fahrtauglichkeit entscheiden. Ferner wurde untersucht, ob es zu einer subjektiven Einschränkung beim Fahren eines Kfz mit Ruhigstellung kam.

Insgesamt zeigt sich ein homogenes Studienkollektiv mit einem ausgeglichenen Geschlechterverhältnis und einer gleichwertigen Verteilung von Ruhigstellungen der oberen und unteren Extremität (Tab. 2). Das durchschnittliche Patientenalter von 39,1 Jahren repräsentiert ein Patientenkollektiv, dass unter dem durchschnittlichen Bevölkerungsalter von 45,9 Jahren (Erfassungsjahr 2020) lag [10]. Die durchschnittliche Fahrleistung von 255,4 km/Woche demonstriert eine regelhafte Nutzung des Pkw. Dieses Patientenkollektiv ist jedoch, allein durch das Alter bedingt, durch z. B. sportliche Aktivität in erhöhtem Maße gefährdet, Extremitätenverletzungen zu erleiden (z. B. Durchschnittsalter für Verletzungen beim Tennissport 43,6 Jahre) [12]. Gleichzeitig kann die Fahrtauglichkeit durch die Ruhigstellung bereits von einem Gelenk eingeschränkt sein [28]. Es fehlen jedoch klare Handlungsempfehlungen, wann die Fahrtauglichkeit bei welcher Bewegungseinschränkung eingeschränkt ist. Für Patienten besteht die Möglichkeit, sich z. B. auf Informationsseiten des „Allgemeinen Deutschen Automobilclub“ (ADAC e. V.) zu erkundigen, jedoch wird auch hier die Empfehlung ausgesprochen, sich beim behandelnden Arzt zu informieren [9]. Eine Entscheidung auf Grundlage der subjektiven Einschätzung der Patienten erscheint ebenfalls nicht sinnvoll, da sich in unserem Kollektiv nur 14,1 % als fahrtauglich einschätzten, jedoch 33,0 % ein Kfz während der Ruhigstellung führten. In einer vorangegangenen Übersichtsarbeit wurde die Einschätzung der Fahrtauglichkeit als schwierig eingeschätzt [13]. Auch in anderen Ländern, z. B. USA, gibt es keine eindeutig definierten Kriterien, wann eine Fahrtauglichkeit („impaired driving function“) aufgrund von Einschränkungen des Bewegungsapparates (ausgenommen Amputationen) nicht mehr gegeben ist, aufgrund fehlender Evidenz [26].

Über die Hälfte der Patienten (55,0 %) gab an (Abb. 2), dass sie den Arzt für die Einschätzung der Fahrtauglichkeit bei einer Ruhigstellung verantwortlich sehen. Rechtlich gesehen ist jedoch der Patient selbst verantwortlich seine Fahrtauglichkeit, insbesondere bei temporärer Veränderung des Gesundheitszustandes, einzuschätzen. und der Arzt soll lediglich beratend zur Seite stehen [8]. Weiterhin wird von der Bundesanstalt für Straßenwesen hervorgehoben, dass für die Begutachtung der Fahrtauglichkeit eine spezielle verkehrsmedizinische Expertise erforderlich ist, sodass der behandelnde Arzt im Regelfall nicht für eine offizielle Begutachtung infrage kommt [8].

Für die dem Arzt vom Patienten übertragene Entscheidungsverantwortung fehlen ebenfalls aktuell klare Handlungsempfehlungen. Verschiedene Arbeitsgruppen haben sich bereits mit der Untersuchung von den erforderlichen muskuloskeletalen Ressourcen zum Führen eines Kfz und ihrer möglichen Einschränkung durch Ruhigstellung oder unfallchirurgisches/orthopädisches Krankheitsbild befasst [5, 7, 14]. Eine vorangegangene Übersichtsarbeit hat für häufige unfallchirurgische/orthopädische Krankheitsbilder Empfehlungen zusammengefasst, jedoch wird limitierend angegeben, dass die Datenlage insbesondere für die obere Extremität unvollständig ist [13]. Auch weitere Übersichtsarbeiten konnten keine eindeutigen Empfehlungen für die Fahrtauglichkeit bei Ruhigstellung der oberen Extremität finden [25, 28].

Die häufigste Untersuchung in der Fahrtauglichkeitsanalyse ist die Untersuchung der Bremskraft und der Bremsreaktionszeit [3, 4, 26]. Dammerer et al. konnten zeigen, dass die bewegungseinschränkende Sprunggelenkorthese einen signifikanten Einfluss auf die Bremsreaktionszeit hatte, während eine Sprunggelenkbandage keinen signifikanten Einfluss hatte [5]. In dem hier untersuchten Studienkollektiv war die häufigste Orthesenart die Sprunggelenkorthese (ohne Limitierung der Extension/Flexion), jedoch führten nur 50,0 % der Patienten während der Ruhigstellung ein Kfz. Da für das Führen eines Kfz komplexe Bewegungsabläufe erforderlich sind, die sich in die 3 erforderlichen körperlichen Ressourcen Kraft, Bewegungsausmaß und Koordination unterteilen lassen, ist die Prüfung der Fahrtauglichkeit herausfordernd und kann nicht alleinig auf Grundlage von Untersuchungen zu Bremskraft und Bremsreaktionszeit beantwortet werden. Vielmehr fehlen aktuell repräsentative Studien, welche das Zusammenspiel der Extremitäten in Form von Bewegungsketten sowie von Kopf/Wirbelsäule darstellen, um ein gesamtheitliches Bild zu berichten. Da die durchschnittliche Ruhigstellungsdauer des Gesamtkollektives fast 6 Wochen betrug, kann eine Fehleinschätzung der Fahrtauglichkeit jedoch auch relevante sozioökonomische Einflüsse haben.

Bezüglich des erforderlichen Bewegungsumfanges zum Führen eines Kfz gibt es bisher nur wenige Literatur: Rawal et al. konnte im Rahmen einer biomechanischen Analyse Normwerte der oberen Extremität definieren, die die Range of Motion beim Führen eines Kfz beschreiben [23]. Latz et al. beschrieben beispielsweise die Range of Motion des Sprunggelenks beim Führen eines Pkw [14]. Nicht untersucht ist jedoch bisher, in welchem Ausmaß bei Ruhigstellung eines Gelenks die anderen Gelenke eine Kompensation durchführen können. In unserem Patientenkollektiv war das am häufigsten ruhiggestellte Gelenk das Handgelenk, jedoch führten nur 17,4 % der Patienten bei Handgelenkruhigstellung ein Kfz. Eine Studie im Fahrsimulator konnte jedoch zeigen, dass eine isolierte Ruhigstellung des Handgelenks keinen Einfluss auf die Lenkfähigkeit und das Fahrverhalten hatte [15].

Bei Betrachtung der Subgruppe, die ein Kfz während einer Ruhigstellung geführt hat, zeigt sich, dass die Verletzungsart mit für die Entscheidungsfindung verantwortlich zu sein scheint. So war der Anteil an frakturbedingten Ruhigstellungen in der Subgruppe deutlich niedriger als im Gesamtkollektiv, sodass die Verletzungsschwere einen Einfluss zu haben scheint. So haben sich Patienten, die eine Bandverletzung oder eine Distorsion erlitten haben, häufiger für das Führen eines Kfz während der Verletzung entschieden (Abb. 3). Dies könnte ein Hinweis sein, dass die eingangs beschriebene eigenverantwortliche Einschätzung der Fahrtauglichkeit von der Verletzungsentität beeinflusst wird. Auch zeigten sich bei den Patienten, die während der Ruhigstellung ein Kfz führten, ein höherer Anteil von Verletzungen der linken Seite. Einzelne Studien konnten zeigen, dass es seitenspezifische Unterschiede gibt, wann ein Kfz, beispielsweise nach einem Kreuzbandersatz, wieder geführt werden kann [18].

Die Patienten, die ein Kfz führten, gaben jedoch zu 61,3 % an, eine subjektive Behinderung durch die Ruhigstellung beim Führen eines Kfz zu verspüren, schätzten sich aber dennoch als fahrtauglich ein (Tab. 3). Ebenfalls gaben 87,5 % ein Sicherheitsgefühl trotz der angegebenen Behinderung während der Fahrt an. Das einzelne Patienten trotz subjektiver Behinderung und teilweise eingeschränktem Sicherheitsgefühl ein Kfz führten, wirft die Frage auf, ob eine andere sozioökonomische Abhängigkeit vom Kfz oder eine erhöhte Risikobereitschaft bestand: Die Risikobereitschaft zeigte sich in der Subgruppe im Vergleich zum Gesamtkollektiv annähernd gleich. Bezüglich der Abhängigkeit vom Kfz wurde sowohl eine höhere Abhängigkeit bezüglich der privaten als auch beruflichen Nutzung angegeben. Frazier et al. führten eine Befragung durch von Patienten nach ambulanten handchirurgischen Eingriffen, wann und aus welchen Beweggründen sie ans Steuer zurückkehrten, und konnte zeigen, dass die Abhängigkeit vom Kfz einen relevanten Einfluss auf eine frühere Rückkehr ans Steuer hat [6].

Abschließend scheint die Getriebeart (Automatik- vs. Schaltgetriebe) keinen relevanten Einfluss auf die Entscheidung bezüglich des Fahrens während einer Ruhigstellung zu haben (Tab. 3). Aktuell ist noch unklar, ob beispielsweise mit einer isolierten Ruhigstellung der linken unteren Extremität das Führen eines Kfz mit Automatikgetriebe möglich wäre.

## Resümee

Insgesamt schätzte das Patientenkollektiv seine Kenntnis über die Gesetzeslage als gering ein und sah den behandelnden Arzt in der Mehrzahl in der Entscheidungsverantwortung. Der Arzt hat jedoch, rechtlich gesehen, nur eine beratende Funktion und trägt nicht die Entscheidungsverantwortung.

Die Patienten, die während der Ruhigstellung ein Kfz führten, zeigten eine höhere private als auch berufliche Abhängigkeit vom Kfz. Gleichzeitig hatte die Verletzungsschwere einen Einfluss auf die Entscheidung. Patienten mit Distorsionen oder Bandverletzungen führten häufiger ein Kfz, verglichen mit Patienten, die aufgrund einer Fraktur behandelt wurden.

Somit ist eine frühe Mobilitätsanamnese durch den behandelnden Arzt entscheidend, um Risikopatienten bezüglich der Fahrt während einer Gelenkruhigstellung zu identifizieren. Weitere Studien sind erforderlich, um insbesondere neben der Bremsreaktionszeit und Bremskraft auch Aussagen auf biomechanischer Ebene hinsichtlich erforderlicher Range of Motion und möglichen Kompensationsmechanismen bei Ruhigstellung eines Gelenks treffen zu können. Für den Arzt, der von der Mehrzahl der Patienten als Entscheidungsverantwortlicher angesehen wird, muss es das Ziel sein, eine bessere Grundlage für die Entscheidung über die Fahrtauglichkeit bei orthopädischen/unfallchirurgischen Erkrankungen zu schaffen.

## Fazit für die Praxis

- Die Kenntnis über die Gesetzeslage hinsichtlich der Fahrtauglichkeit ist gering.
- Die private und berufliche Abhängigkeit vom Kfz ist ein entscheidender Faktor, ob ein Kfz bei Ruhigstellung geführt wird.
- Patienten mit schwereren Verletzungen vermeiden das Führen eines Kfz während der Ruhigstellung.

## Data Availability

Die erhobenen Datensätze können auf begründete Anfrage in anonymisierter Form beim korrespondierenden Autor angefordert werden. Die Daten befinden sich auf einem Datenspeicher am Universitätsklinikum Düsseldorf in der Klinik für Orthopädie und Unfallchirurgie.
